# Cardiovascular magnetic resonance imaging to assess myocardial fibrosis in valvular heart disease

**DOI:** 10.1007/s10554-017-1195-y

**Published:** 2017-06-22

**Authors:** Tomaz Podlesnikar, Victoria Delgado, Jeroen J. Bax

**Affiliations:** Department of Cardiology, Heart and Lung Center, Leiden University Medical Center, Albinusdreef 2 2333 ZA, Leiden, The Netherlands

**Keywords:** Cardiovascular magnetic resonance, Valvular heart disease, Mitral regurgitation, Aortic stenosis, Aortic regurgitation, Late gadolinium enhancement, T1 mapping, Tagging, Feature tracking

## Abstract

The left ventricular (LV) remodeling process associated with significant valvular heart disease (VHD) is characterized by an increase of myocardial interstitial space with deposition of collagen and loss of myofibers. These changes occur before LV systolic function deteriorates or the patient develops symptoms. Cardiovascular magnetic resonance (CMR) permits assessment of reactive fibrosis, with the use of T1 mapping techniques, and replacement fibrosis, with the use of late gadolinium contrast enhancement. In addition, functional consequences of these structural changes can be evaluated with myocardial tagging and feature tracking CMR, which assess the active deformation (strain) of the LV myocardium. Several studies have demonstrated that CMR techniques may be more sensitive than the conventional measures (LV ejection fraction or LV dimensions) to detect these structural and functional changes in patients with severe left-sided VHD and have shown that myocardial fibrosis may not be reversible after valve surgery. More important, the presence of myocardial fibrosis has been associated with lesser improvement in clinical symptoms and recovery of LV systolic function. Whether assessment of myocardial fibrosis may better select the patients with severe left-sided VHD who may benefit from surgery in terms of LV function and clinical symptoms improvement needs to be demonstrated in prospective studies. The present review article summarizes the current status of CMR techniques to assess myocardial fibrosis and appraises the current evidence on the use of these techniques for risk stratification of patients with severe aortic stenosis or regurgitation and mitral regurgitation.

## Introduction

Valvular heart disease (VHD) is an important public-health problem with an increasing prevalence along with ageing of the population [1]. Moderate and severe VHD on echocardiography affects 2.5% of the population of the United States and increases up to 11.7% in the group of patients aged 75 and older [2]. The decision to operate in patients with severe VHD is frequently complex and relies on an individual risk–benefit analysis. In general, improvement in prognosis compared with natural history of the disease should outweigh the risk of intervention and its potential late consequences, particularly prosthesis-related complications. Current guidelines recommend to intervene in patients with symptomatic severe VHD and in asymptomatic patients with reduced left ventricular (LV) ejection fraction, LV dilatation, pulmonary hypertension, right ventricular dilatation and dysfunction and presence of atrial fibrillation [1, 3]. However, most of these adverse consequences of severe VHD are observed in advanced stages of the disease and are partially irreversible after intervention, leading to suboptimal long-term clinical outcomes [4]. Therefore, additional markers that identify early structural and functional consequences of severe VHD before irreversible damage of the myocardium occurs would help to redefine the optimal timing for intervention.

Chronic pressure and volume overload caused by severe left-sided VHD results in LV remodeling. Changes in the extracellular matrix with deposition of collagen I and loss of myofibers at a later stage result in myocardial fibrosis, the hallmark of LV remodeling [5, 6]. Cardiovascular magnetic resonance (CMR) imaging techniques permit direct and indirect assessment of myocardial fibrosis. T1 mapping and late gadolinium enhancement (LGE) permit myocardial tissue characterization and provide measures of direct myocardial fibrosis whereas CMR tagging and feature tracking CMR allow for assessment of myocardial deformation (strain), a functional parameter that indirectly reflects myocardial fibrosis. In addition, advances in molecular CMR imaging provide high-specificity tools for detection of myocardial fibrosis. This article provides an overview of current CMR techniques to assess myocardial fibrosis in patients with left-sided VHD.

## CMR techniques for direct assessment of myocardial fibrosis

LV remodeling in response to chronic pressure and volume overload caused by VHD is characterized by progressive increase of the interstitial space with increased collagen volume fraction (reactive fibrosis) and eventually apoptosis of myocardial cells which are replaced by firm fibrous tissue (replacement fibrosis or scar). T1 mapping and LGE CMR techniques are currently the most frequently used techniques to directly assess myocardial fibrosis (Table 1).

**Table 1:** Cardiovascular magnetic resonance techniques to assess myocardial fibrosis valvular heart disease

CMR technique	Availability	Fibrosis specificity	Advantages	Limitations	Experience in VHD
T1 mapping (native T1 and ECV quantification)	++	+++	Assessment of diffuse fibrosis, early disease changes (preclinical stages). Quantification of the degree of fibrosis	Multiple methodologies, no standardized reference values, overlap between normal and diseased myocardium	++
Late gadolinium enhancement	+++	+++	Reference standard for assessment of replacement fibrosis	Focal fibrosis assessment only	+++
Molecular imaging	±	++++	Improved visualization of fibrosis, investigation of underlying processes (necrosis, apoptosis, inflammation, scar maturation…)	Experimental technique, animal studies only	–
CMR tagging	++	+	Current gold standard for myocardial deformation assessment, high reproducibility of the results	Expertise, additional scan sequences, time consuming post-processing, tag fading through cardiac cycle (only with some techniques), limited in assessment of thin myocardium	++
Feature tracking CMR	+++	+	Post-processing of SSFP cines (no additional scan sequences), relatively fast post-processing, high feasibility	Susceptible to through-plane motion artifacts, limited inter-vendor agreement	+

*CMR* cardiovascular magnetic resonance, *ECV* extracellular volume, *SSFP* steady state free precession, *VHD* valvular heart disease

### CMR T1 mapping

**Fig. 1:**
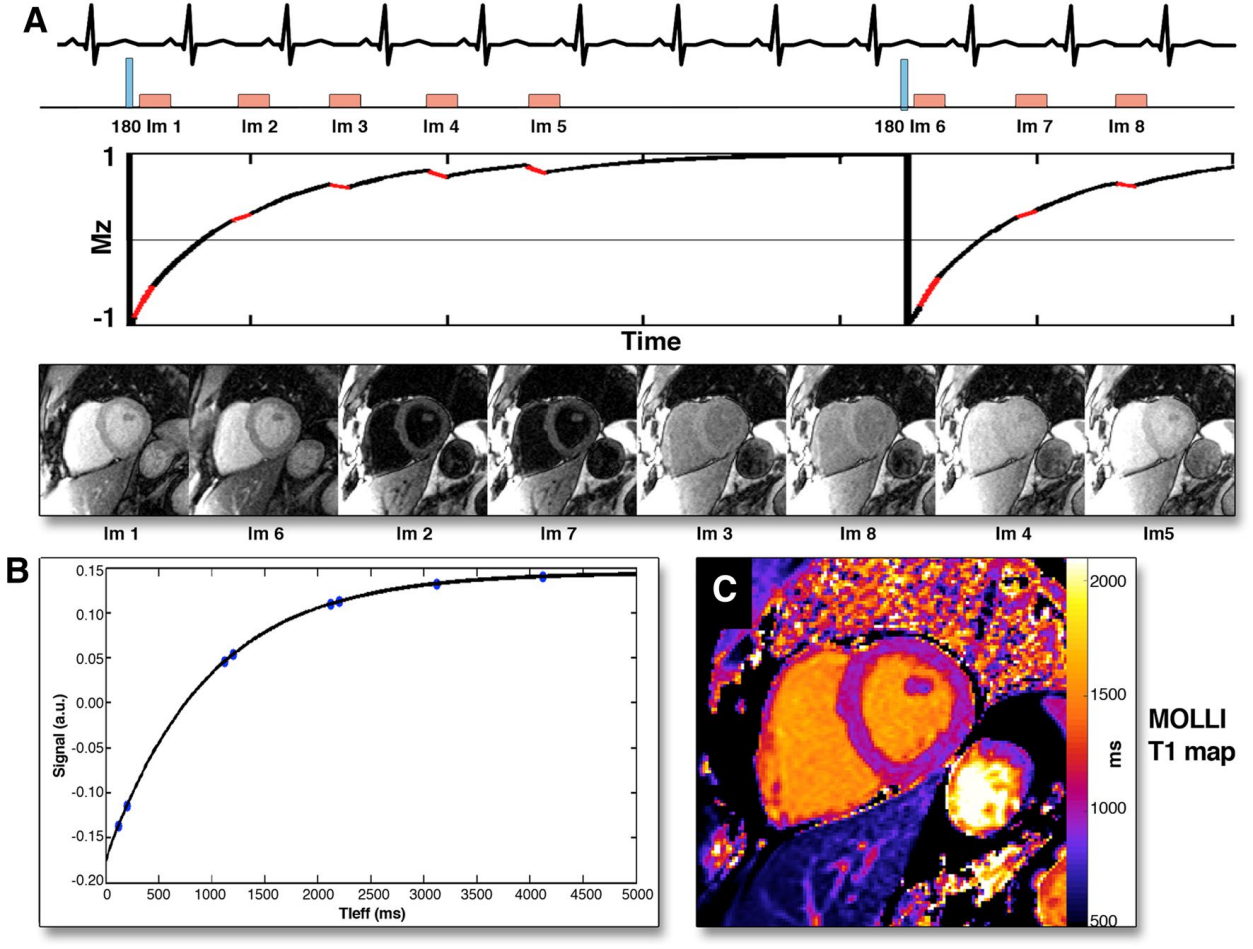
Modified Look-Locker (MOLLI) technique for myocardial T1 mapping. After radiofrequency inversion pulse, myocardial tissue longitudinal magnetization in a stable magnetic field returns to the equilibrium and a series of images are acquired in diastole over several heart beats (**A**). The images are sorted in order of increasing T1 times and the T1 recovery curve is obtained by plotting respective signal intensities against T1 time (**B**). The T1 map is obtained by applying this technique for all pixels in the image (**C**). Reproduced with permission from Taylor et al. [7]

The longitudinal magnetization relaxation time of the myocardium, so-called T1 time, is highly sensitive to processes that increase the interstitial space and can be quantified with various techniques [7]. One of the most commonly used in clinical practice is the modified Look-Locker pulse sequence where multiple single-shot images are acquired intermittently in diastole during 9–17 cardiac cycles and the inversion recovery curves are generated (Fig. 1, panels A and B). The T1 time can be obtained for any myocardial segment and T1 maps can be generated by determining the T1 time at each pixel location (Fig. 1, panel C). Three T1 mapping-derived metrics have been proposed as markers of increased myocardial fibrosis: the native T1 time, the post-contrast T1 time and the myocardial extracellular volume (ECV). With the increase of interstitial fibrosis, the native T1 values (without the use of gadolinium contrast) become longer whereas the post-contrast T1 values become shorter. By combining them, myocardial ECV fraction can be computed, which quantifies the extracellular matrix space. In the absence of amyloid deposition or edema, collagen I is the main component of the extracellular matrix space and therefore the myocardial ECV fraction is considered a robust marker of myocardial fibrosis [8–10]. The added value of these metrics over LGE is the ability to quantify the degree of fibrosis and, particularly, to detect diffuse interstitial fibrosis, often associated with early stages of the disease.

However, it should be noted that the cut-off values of the T1 mapping-derived metrics to define fibrosis cannot be currently established since the values show considerable overlap in normal and diseased myocardium [11]. Moreover, neither of the techniques is entirely specific to myocardial fibrosis; abnormal myocardial ECV fraction can be observed in infiltrative diseases (i.e., amyloidosis) and edema, while native T1 values may also be altered in iron deposition and diffuse fat infiltration [12]. Furthermore, standardization of CMR T1 mapping techniques is necessary to obtain reproducible measurements across different vendors and institutions.

### Late gadolinium contrast enhanced CMR

**Fig. 2:**
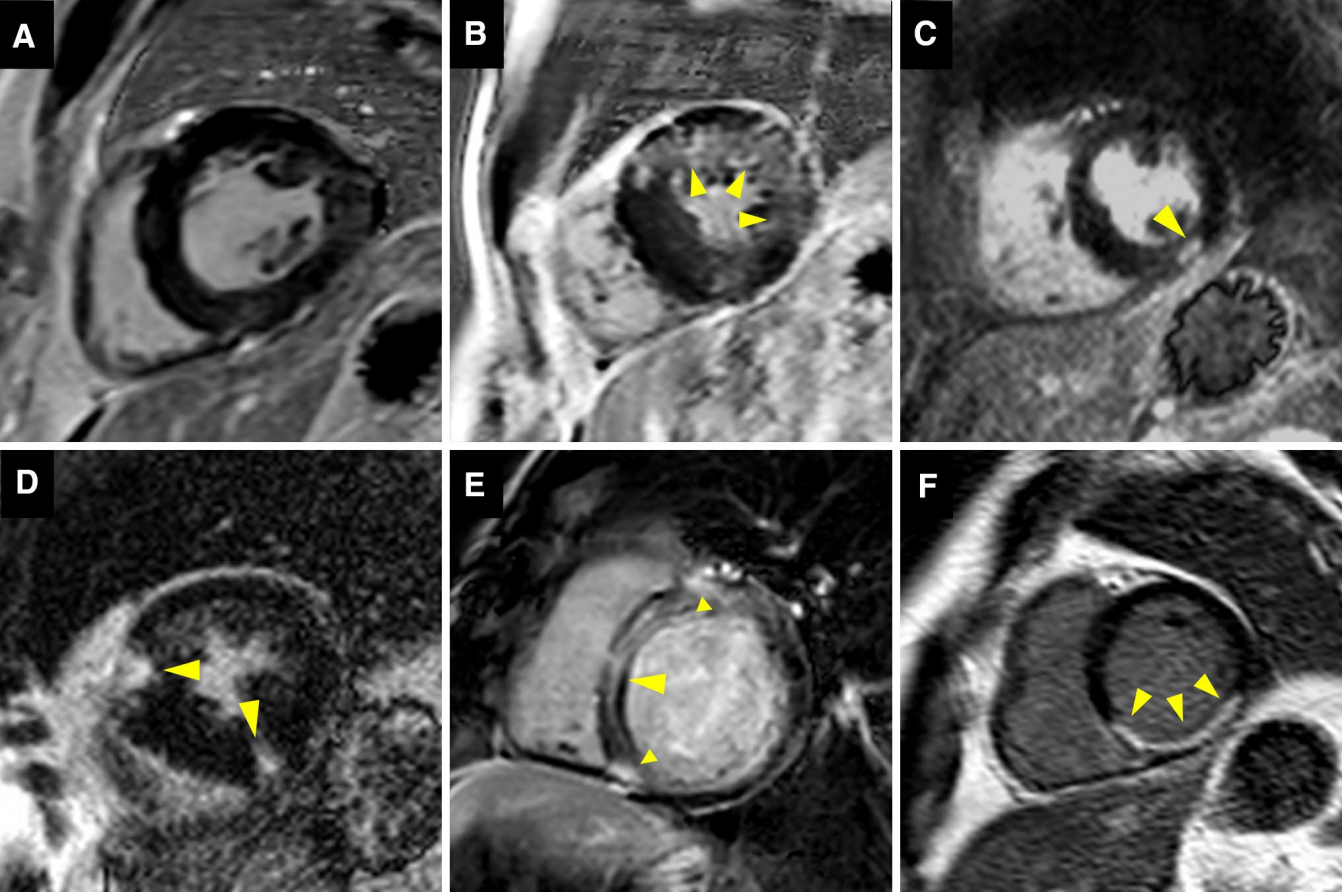
Patterns of late gadolinium enhancement (LGE). **A** shows no LGE, no focal replacement fibrosis. **B**–**E** demonstrate different patterns of non-infarct myocardial fibrosis: **B** diffuse patchy LGE of the anterior and lateral wall (*arrows*); **C** focal nodular LGE of the inferior wall (*arrow*); **D** focal LGE of the anterior and inferior right ventricular insertion points (*arrows*) and **E** linear midwall septal LGE with additional foci at the right ventricular insertion points (*arrows*). In **F**, typical infarct-type subendocardial LGE distribution is shown (*arrows*)

LGE CMR is considered the reference standard to quantify myocardial replacement fibrosis and scar. The increased extracellular space and decreased capillary density of the fibrous tissue result in increased volume of distribution and prolonged wash-out of gadolinium in comparison to the normal myocardium [13]. 10–20 min after intravenous administration of gadolinium, inversion recovery images are acquired in mid to late diastole. The inversion time is chosen to null the normal myocardium and provide the best tissue contrast between fibrous tissue, which appears bright, and normal myocardium, which appears black. Distinct patterns of LGE have been described in various cardiac diseases and associated with adverse prognosis [14–19] (Fig. 2).

### Molecular magnetic resonance imaging

Molecular magnetic resonance imaging with the use of collagen-specific contrast agents is a new experimental method for the assessment of myocardial fibrosis. These novel contrast agents have shown to improve visualization of scar and perfusion defects in animal models of myocardial infarction [20, 21]. Furthermore, an elastin/tropoelastin-targeting contrast agent has provided interesting insights into the pathophysiology of remote myocardium extracellular matrix remodeling in a mice model of acute myocardial infarction [22]. Several other molecular probes have been synthesized to study individual processes involved in fibrosis formation, like necrosis, apoptosis, inflammation and scar maturation [23]. Further efficacy and safety studies are needed before clinical implementation. However, the current evidence is promising for future improvements in fibrosis detection and monitoring of molecular processes associated with myocardial remodeling.

## CMR techniques for indirect assessment of myocardial fibrosis

The functional consequences of myocardial fibrosis such as increased LV stiffness, impaired LV diastolic and systolic function, can be evaluated with CMR tagging and feature tracking CMR (Table 1). These techniques evaluate the active deformation (strain) of the myocardium in three orthogonal directions: radial, circumferential and longitudinal. In patients with VHD, the measurement of LV ejection fraction, which merely reflects the change in LV volumes between systole and diastole, may be misleading. For example, in patients with mitral regurgitation, LV ejection fraction may be preserved for long time since the LV is emptying in a low-pressure chamber (left atrium) while myocardial longitudinal strain may be impaired [24]. In patients with severe aortic stenosis, the LV hypertrophy, developed in response to the pressure overload, reduces the wall stress and maintains the LV ejection fraction. However, myocardial longitudinal strain may be impaired [25]. CMR tagging and feature tracking CMR track distinctive features of the myocardium throughout the cardiac cycle and calculate mechanical indices, such as strain, strain-rate, twist and torsion.

### CMR tagging

This method is based on alteration of the myocardial tissue magnetization to create trackable markers within the myocardium which are visualized as dark lines in the form of a grid pattern. This allows immediate visual assessment of myocardial deformation, but for a more objective approach and quantification additional post-processing is employed. Recent developments in pulse sequences and image processing have resulted in a plethora of new tagging techniques [26]. The main advantage of CMR tagging over feature tracking CMR is that the imposed tags are more clearly defined and easier tracked than the natural features and are not subjected to through plane displacements, thereby providing more reproducible measurements [27]. The main shortcomings of this technique are the need for additional, elaborate scan sequences with limited accuracy when applied to thin myocardium (such as the remodeled, thinned-wall LV, the right ventricle and the atria) and the time-consuming post-processing.

### Feature tracking CMR

**Fig. 3:**
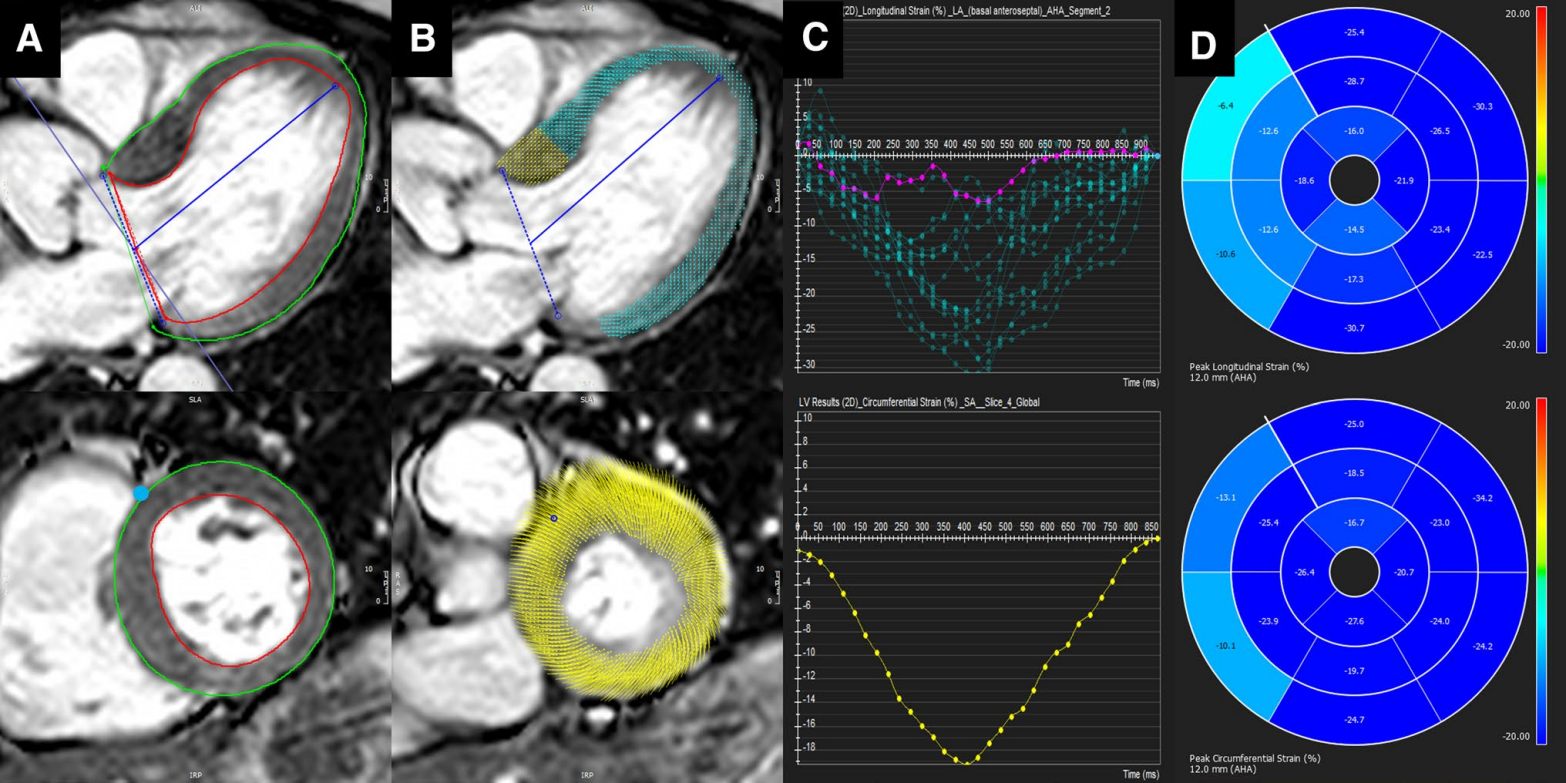
Feature tracking cardiovascular magnetic resonance (CMR) in a patient with severe aortic stenosis. **A** Long-axis (*top*) and a mid-cavity short-axis (*bottom*) end-diastolic steady state free precession images. Left ventricular endo- and epicardium are contoured (*red* and *green lines*) and the anterior right ventricular insertion point is marked in short-axis (*blue dot*). **B** Fully automated feature tracking analysis is performed by tracking distinctive features along the outlined myocardium borders. **C** The derived time-strain curves show a wide variation in segmental longitudinal strain (*top*) and normal global peak circumferential strain (*bottom*). The *purple colored curve* corresponds to the anteroseptal segment. **D** The 16-segment bullseye plots for longitudinal (*top*) and circumferential (*bottom*) left ventricular strain, showing impaired myocardial deformation of the basal interventricular septum. (Feature tracking analysis was performed with cvi^42^ v5.3, *Circle* Cardiovascular Imaging, Calgary, Canada)

Feature tracking CMR is based on post-processing of standard steady state free precession cine images, similar to echocardiographic speckle tracking. Feature tracking CMR algorithms focus on the endo- and epicardial borders and detect the in- and outward motion of the cavity-tissue interface [27, 28]. Global and segmental LV longitudinal, circumferential and radial strain, strain-rates, and LV rotational mechanics can be derived from standard long- and short-axis views (Fig. 3). Global rather than segmental strain values appear the most reproducible [29–31]. Additional methodology standardization is an important prerequisite for wider dissemination of this technique in clinical practice.

## CMR left ventricular myocardial fibrosis assessment in VHD: clinical evidence

Accumulating evidence on the deleterious impact of LV myocardial fibrosis on clinical outcomes after surgical treatment of left-sided VHD has raised interest on tissue characterization and LV strain with CMR techniques [19, 32–36]. This evidence is summarized for aortic stenosis (AS) and regurgitation (AR) and for mitral regurgitation (MR) in the following sections.

### Aortic stenosis

The pressure overload caused by AS increases LV wall stress and as a consequence the myocardium responds with myocyte hypertrophy to maintain LV systolic function. This myocardial hypertrophy is characterized by an increased muscle fiber diameter with parallel addition of new myofibrils [37]. Furthermore, there is an increase of interstitial fibrosis and myocyte apoptosis, partially as a consequence of oxygen supply–demand mismatch and myocardial ischemia [37–39]. At a late stage in the natural history of severe AS, the LV myocardium is characterized by large areas of myocyte loss and replacement fibrosis causing LV systolic dysfunction and associated with poor prognosis [38].

**Fig. 4:**
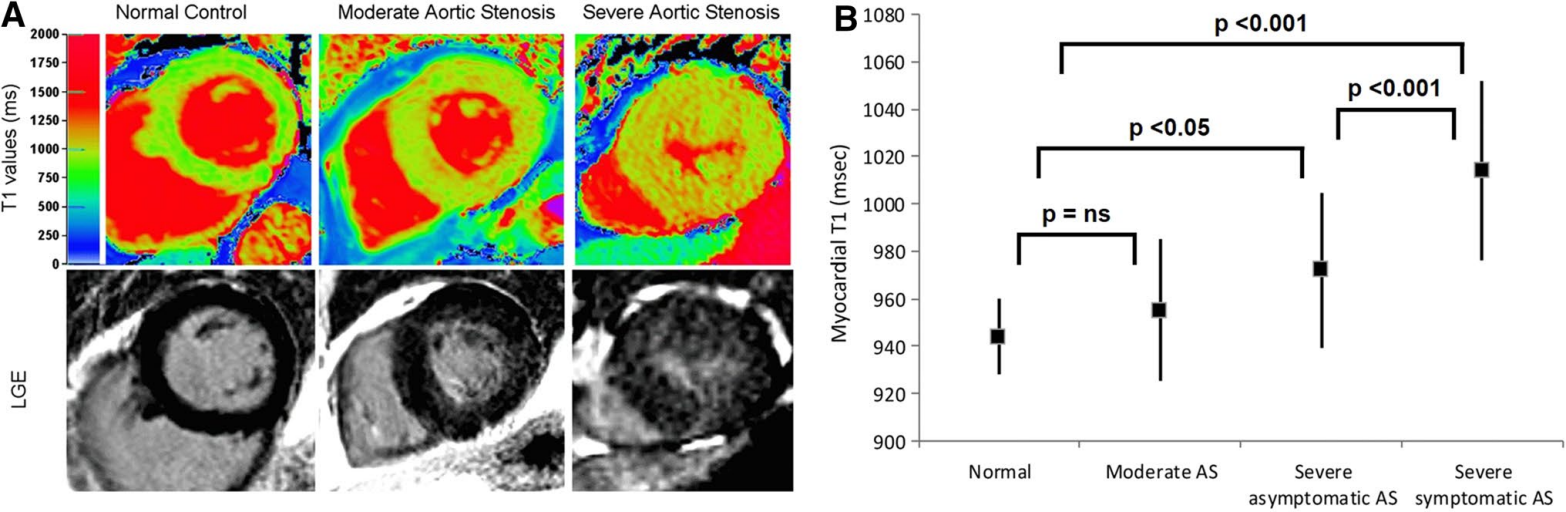
Native T1 mapping in aortic stenosis. **A** Color maps of T1 values of mid-ventricular short-axis slices (*top row*) and corresponding LGE images (*bottom row*) of normal controls and patients with moderate and severe AS. The *left column* shows a normal volunteer (T1 = 944 ms), the middle column a patient with moderate AS and moderate left ventricular hypertrophy (T1 = 951 ms) and the *right column* shows a patient with severe AS with severe left ventricular hypertrophy (T1 = 1020 ms). **B** *Whisker-plots* of myocardial T1 values of normal controls and of patients with moderate AS, asymptomatic severe AS and symptomatic severe AS. The between-group comparisons with the corresponding p-values are also presented. *AS* aortic stenosis, *LGE* late gadolinium enhancement, *ns* non-significant. Adapted with permission from Bull et al. [40]

**Fig. 5:**
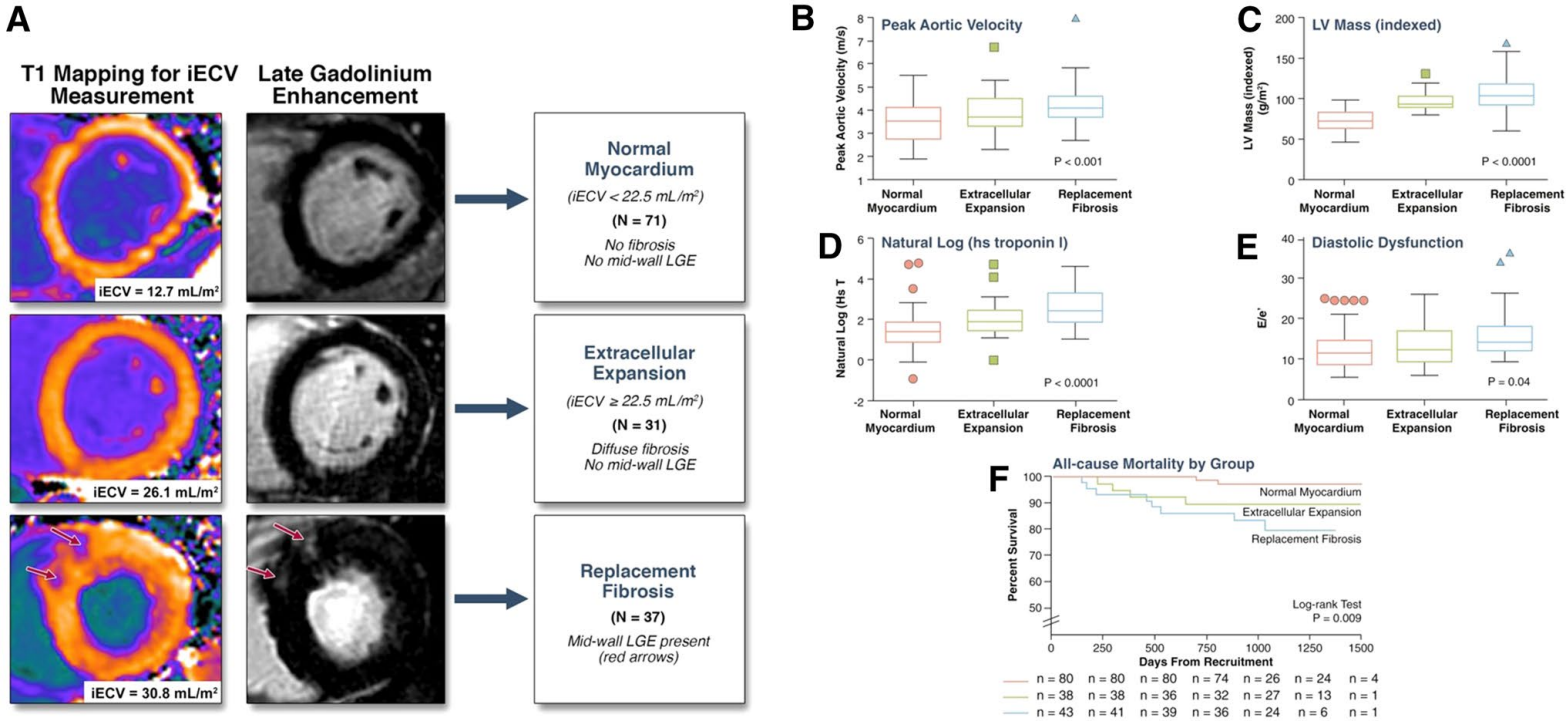
Prognostic implications of interstitial and replacement fibrosis in aortic stenosis. **A** Patients with mild to severe aortic stenosis were categorized into three groups based upon cardiovascular magnetic resonance assessments of myocardial fibrosis: normal myocardium [indexed extracellular volume (iECV) < 22.5 ml/m^2^, no late gadolinium enhancement (LGE)], diffuse myocardial fibrosis (iECV ≥ 22.5 ml/m^2^, no LGE) and replacement fibrosis (presence of midwall LGE). There was a stepwise increase in: **B** severity of valve narrowing; **C** degree of left ventricular (LV) hypertrophy; **D** myocardial injury, assessed by high-sensitivity troponin I concentration (hsTni); **E** LV diastolic dysfunction; and **F** all-cause-mortality with increased diffuse myocardial fibrosis and replacement fibrosis. Adapted with permission from Chin et al. [34]

The early changes in the interstitial space with increased deposition of collagen I can be assessed with CMR T1 mapping (Table 2) [8, 34, 40–46]. Several studies have validated LV native T1 values and myocardial ECV fraction against histology in patients with AS undergoing aortic valve replacement [8, 34, 40, 41]. In 109 patients with moderate and severe AS, Bull and colleagues showed that LV native T1 values were significantly higher among patients with symptomatic severe AS compared with moderate and asymptomatic severe AS (1014 ± 38 vs. 955 ± 30 and 972 ± 33 ms, respectively; p < 0.05) (Fig. 4) [40]. A significant correlation was observed between native T1 values and collagen volume fraction assessed on myocardial biopsies (R = 0.65, p = 0.002). Similarly, Flett and coworkers validated the measurement of myocardial ECV fraction in 18 patients with severe AS [8]. ECV strongly correlated with the histological collagen volume fraction (R^2^ = 0.86; p < 0.001). Although still not implemented in routine clinical practice, the measurement of myocardial ECV in patients with AS has important clinical implications [34, 43–46]. Increased ECV has been associated with symptoms, worse LV systolic and diastolic function, higher levels of cardiac troponin T and ECG strain [34, 43–46]. Recently, Chin et al. reported the prognostic implications of myocardial ECV fraction corrected for LV end-diastolic myocardial volume normalized to the body surface area (iECV) in 166 patients with mild to severe AS [34]. Patients with increased myocardial iECV (≥22.5 ml/m^2^) but without LGE (replacement fibrosis) showed significantly higher all-cause mortality and AS-related mortality rates (36 per 1000 patients-year for both) as compared to the patients with normal myocardium (iECV < 22.5 ml/m^2^, 8 and 0 deaths/1000 patient-years) (Fig. 5).

**Table 2:** CMR studies to detect myocardial fibrosis in valvular heart disease

Study	No. of patients	Valve disease	CMR technique	Main findings
Bull et al. [40]	109	AS	Native T1 mapping	Native T1 values increased along with hemodynamic severity of AS and correlated with the degree of biopsy-quantified fibrosis (R = 0.65; p = 0.002; N = 23)
Lee et al. [41]	80	AS	Native T1 mapping	Native T1 values at 3T CMR were significantly longer in asymptomatic patients with moderate to severe AS compared to normal controls
Flett et al. [8]	18	AS	ECV	ECV correlated strongly with collagen volume fraction on histology (R^2^ = 0.86; p < 0.001)
Dusenbery et al. [44]	35	AS	ECV	ECV was significantly higher in patients with congenital AS than in normal subjects
Flett et al. [43]	66	AS	ECV	Patients with severe AS had higher ECV than normal controls
Chin et al. [34]	166	AS	iECV, LGE	Increased iECV was associated with increased all-cause mortality compared to patients with normal iECV (36 vs. 8 deaths/1000 patient-years, respectively)
Chin et al. [45]	122	AS	ECV, LGE	ECV and percent of midwall replacement fibrosis (LGE) were associated with increased high-sensitivity cardiac troponin I levels
Shah et al. [46]	102	AS	ECV, LGE	LGE and ECV were associated with ECG strain in patients with mild to severe AS
Debl et al. [47]	22	AS	LGE	LGE was associated with severe LV hypertrophy
Rudolph et al. [48]	21	AS	LGE	LGE was associated with increased LV mass index and LV end-diastolic volume index. LGE was not associated with the severity of AS
Dweck et al. [19]	143	AS	LGE	Midwall fibrosis on LGE CMR was associated with higher mortality than infarct-type LGE (HR 8.59; 95% CI 1.97–37.38; p = 0.004 and HR 6.46; 95% CI 1.39-30.00; p = 0.017, respectively)
Barone-Rochette et al. [32]	154	AS	LGE	LGE was an independent predictor of all-cause and cardiovascular mortality in patients with severe AS undergoing surgical valve replacement (HR for all-cause mortality: 2.8; 95% CI 1.3–6.9; p = 0.025)
Weidemann et al. [49]	58	AS	LGE	The extent of LGE in patients with symptomatic severe AS undergoing aortic valve surgery correlated with biopsy-quantified myocardial fibrosis and remained unchanged at 9 months after surgery
Azevedo et al. [33]	54	AS + AR	LGE	LGE correlated with the extent of fibrosis on histology (r = 0.69, p < 0.001) and demonstrated significant inverse correlation with the LVEF improvement after surgery (r=-0.47, p = 0.02) LGE was associated with worse long-term survival (Chi square = 5.85; p = 0.02)
Singh et al. [51]	174	AS	LGE	Patients with asymptomatic moderate and severe AS who presented with valve related complications during follow-up showed comparable extent of LGE than patients who remained asymptomatic
Schneeweis et al. [54], Singh et al. [55]	30, 18	AS	CMR tagging, feature tracking CMR	Reasonable agreement between both techniques, but feature tracking CMR yielded higher strain values than CMR tagging
Mahmod et al. [57]	39	AS	CMR tagging	Patients with AS had impaired LV strain compared to controls
Al Musa et al. [56]	42	AS	CMR tagging, feature tracking CMR	Longitudinal strain rate was impaired in symptomatic vs. asymptomatic patients with severe AS and preserved LVEF (−83.4 ± 24.8%/s and − 106.3 ± 43.3%/s, respectively; P = 0.048)
Musa et al. [36]	98	AS	CMR tagging	Impaired mid-LV circumferential strain was associated with all-cause mortality after aortic valve replacement (HR 1.03; 95% CI 1.01–1.05; p = 0.009)
Meyer et al. [58]	44	AS	Feature tracking CMR	Peak systolic LV strain of the apical segments was significantly impaired in transapical versus transfemoral transcatheter aortic valve replacement
Sparrow et al. [62]	8	AR	T1 mapping	Post-contrast T1 values in abnormally contracting segments were prolonged compared to controls (532 vs. 501 ms, respectively; p = 0.002)
de Meester de Ravenstein [63]	9	AR	ECV	ECV measured on 3T CMR was strongly correlated with the extent of interstitial fibrosis on histology in patients with severe AR (r = 0.79, p = 0.011)
Pomerantz et al. [64]	14	AR	Myocardial tagging	Global longitudinal and circumferential strain were decreased 2 years after aortic valve replacement, despite an improvement in LVEF and LV size
Ungacta et al. [65]	8	AR	Myocardial tagging	Posterior wall circumferential strain was decreased 6 months after surgery
Edwards et al. [68]	35	MR	ECV, native T1 mapping, LGE	Patients with moderate to severe primary MR had higher ECV compared to controls (0.32 ± 0.07 vs. 0.25 ± 0.02, respectively; p < 0.01)
Han et al. [69]	25	MR	LGE	LGE of the papillary muscles was present in 63% of patients with MV prolapse
Chaikriangkrai et al. [35]	48	MR	LGE	The presence of LV LGE in chronic severe MR was associated with worse clinical outcomes (HR 4.8; 95% CI 1.1–20.7; p = 0.037)
Maniar et al. [70]	15	MR	CMR tagging	Patients with chronic moderate and severe MR and preserved LVEF had impaired septal LV strain values compared to normal controls
Mankad et al. [71]	7	MR	CMR tagging	Patients with severe MR and preserved LVEF had reduced circumferential strain compared to controls (12 ± 6 vs. 21 ± 6%, respectively; p ≤ 0.001)
Ahmed et al. [72], Schiros et al. [73], Ahmed et al. [74]	27 35 22	MR	CMR tagging	Global longitudinal and circumferential strain parameters were decreased after MV repair

*AS* aortic stenosis, *AR* aortic regurgitation, *CMR* cardiovascular magnetic resonance, *ECV* extracellular volume, *HR* hazard ratio, *ICU* intensive care unit, *iECV* indexed extracellular volume, *LGE* late gadolinium enhancement, *LV* left ventricle, *LVEF* left ventricular ejection fraction, *MR* mitral regurgitation

LGE, myocardial replacement fibrosis, is detected in 19–62% of patients with severe AS [19, 32, 47, 48]. Two forms of LGE can be observed: the ischemic and the non-ischemic pattern. The ischemic pattern is characterized by subendocardial LGE along specific coronary artery territories whereas in the non-ischemic pattern the distribution of LGE can be diffuse, (multi)focal or linear, confined or patchy, and is predominantly located in the midwall myocardial layer and does not correspond to a specific coronary artery territory (Fig. 2) [19, 32, 47, 48]. The presence and the extent of LGE have been associated with increased LV mass, worse LV ejection fraction, the presence of symptoms, markers of myocardial injury such NT-pro-brain natriuretic peptide and high-sensitivity cardiac troponins and ECG strain (Table 2) [19, 32, 45, 46, 48, 49]. However, LGE was not significantly associated with transaortic gradients or the aortic valve area, common indices of AS severity [19, 32, 48], suggesting that there is different individual susceptibility to develop LV hypertrophy and myocardial fibrosis, likely influenced by multiple factors such as advanced age, male sex, obesity and certain genetic variants [50].

**Fig. 6:**
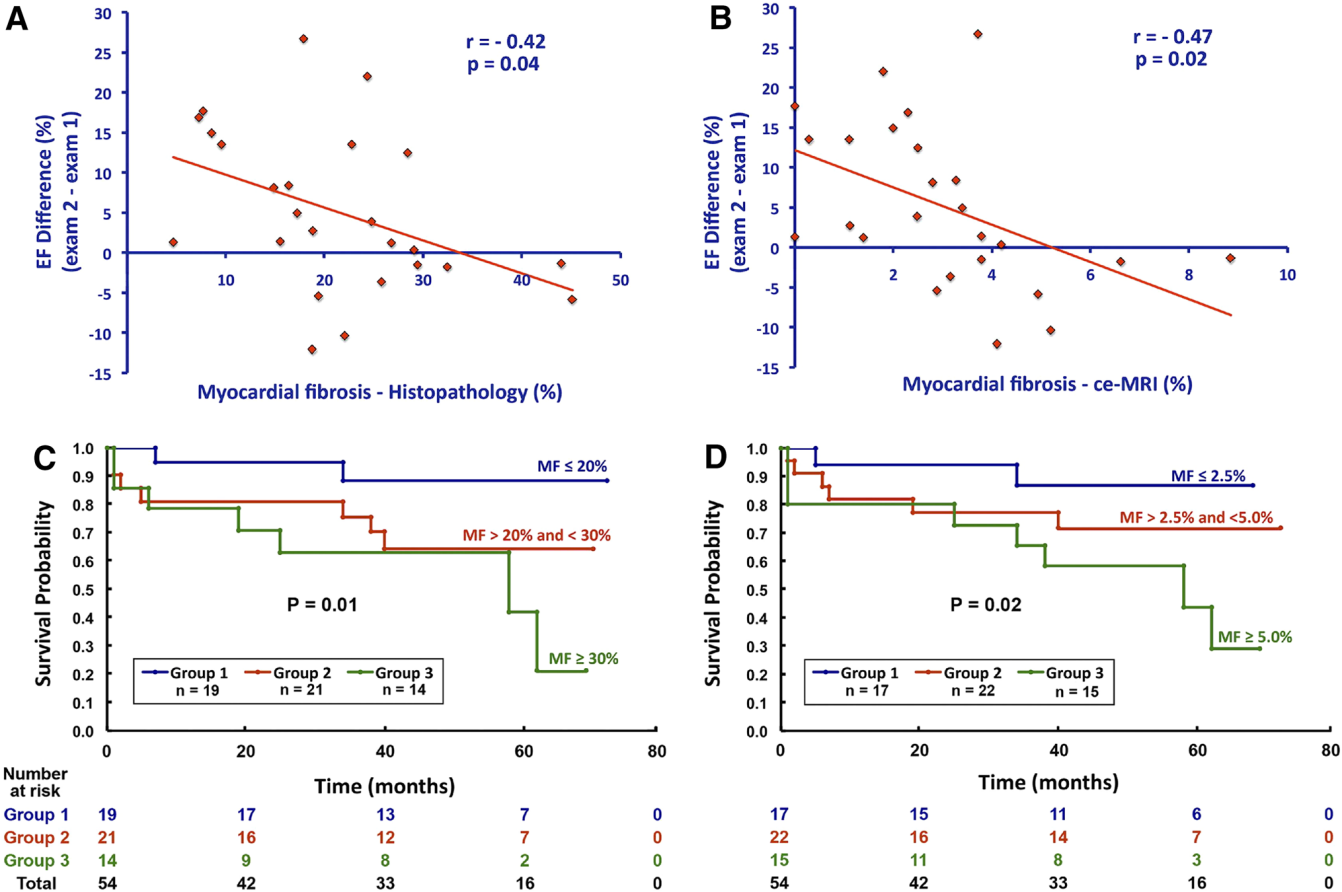
Prognostic implications of late gadolinium enhancement (LGE) cardiovascular magnetic resonance (CMR) in patients with severe aortic stenosis and aortic regurgitation after aortic valve replacement surgery. Linear regression graphs illustrate the inverse relationship between the degree of left ventricular ejection fraction improvement and the amount of myocardial fibrosis by histopathology (**A**) and by LGE CMR (**B**). The Kaplan–Meier graphs demonstrate significantly worse survival after aortic valve replacement in patients with larger myocardial fibrosis assessed by histopathology (**C**) or LGE (**D**). Reproduced with permission from Azevedo et al. [33] *ce-MRI* contrast-enhanced magnetic resonance imaging, *EF* ejection fraction, *MF* myocardial fibrosis

In addition, LGE is an important prognostic marker in patients with AS [19, 32, 33]. In 143 patients with moderate and severe AS who were followed for 2.0 ± 1.4 years, the presence of LGE was associated with an increase in all-cause and cardiac mortality (every 1% increase in LGE mass was associated with 5% increased risk of all-cause mortality; p = 0.005) [19]. When dividing the population according to the pattern of LGE, patients with midwall fibrosis (N = 54) had higher mortality than patients with infarct-type LGE (N = 40) (HR 8.59; 95% CI 1.97–37.38; p = 0.004 and HR 6.46; 95% CI 1.39–30.00; p = 0.017, respectively). Furthermore, in 154 patients with severe AS undergoing surgical aortic valve replacement, the presence of LGE was an independent predictor of all-cause and cardiovascular mortality (HR for all-cause mortality: 2.8; 95% CI 1.3–6.9; p = 0.025) [32]. Importantly, after aortic valve replacement, LGE does not completely regress and has been associated with incomplete LV functional recovery, worse New York Heart Association functional class and worse survival (Fig. 6) [32, 33, 49]. However, detection of LV myocardial fibrosis in patients with asymptomatic moderate and severe AS seems insufficient to identify the patients who will present valve related complications. In the prognostic importance of microvascular dysfunction in aortic stenosis (PRIMID AS) study, including 174 patients with asymptomatic moderate to severe AS, the group of patients who presented with cardiovascular death, major adverse cardiovascular events and development of typical AS symptoms, necessitating referral for aortic valve replacement, showed comparable extent of LGE than patients who remained asymptomatic or free of valve related complications during follow-up [51].

**Fig. 7:**
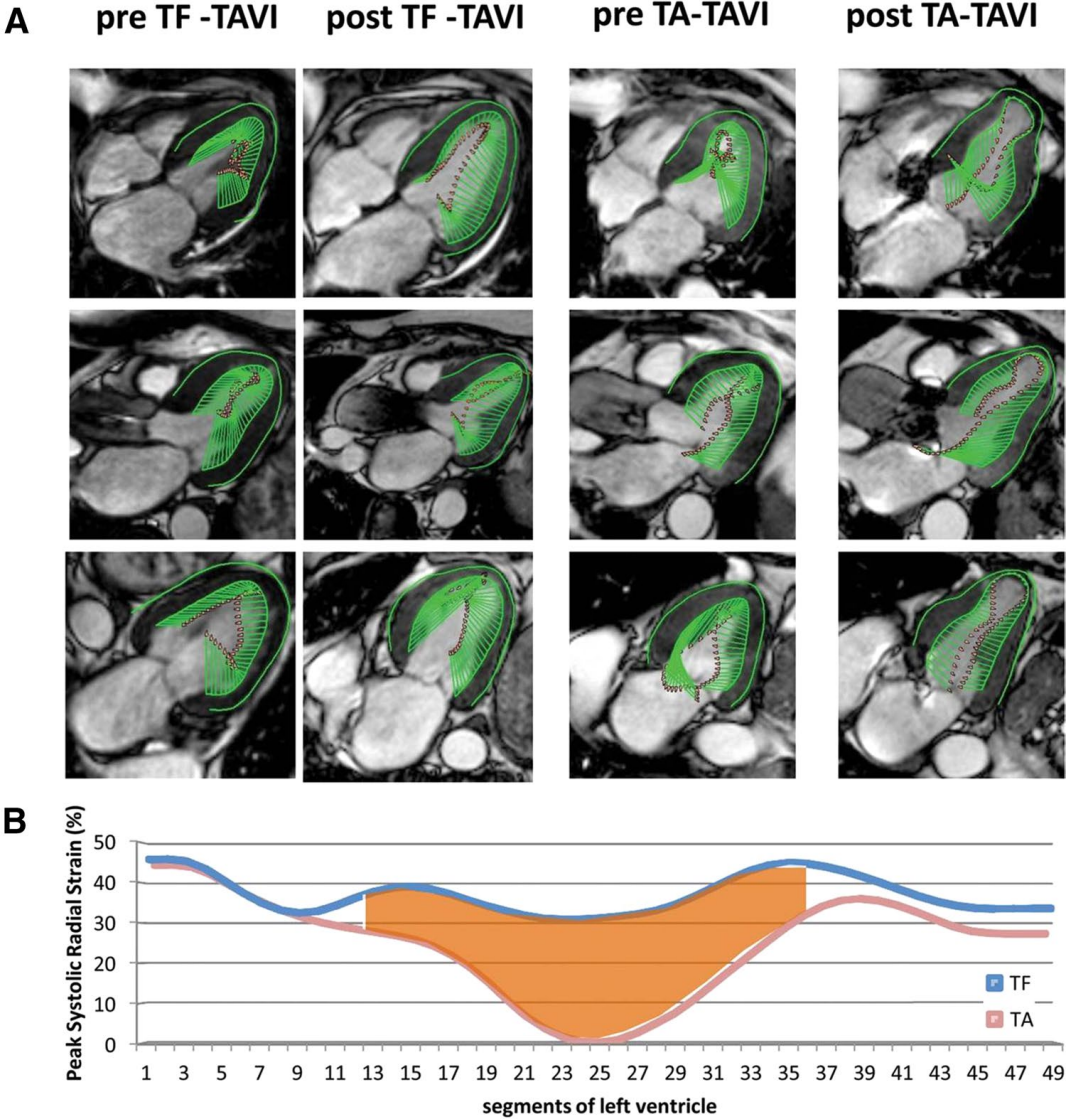
The impact of transcatheter aortic valve implantation on the left ventricular (LV) mechanics, assessed with feature tracking cardiovascular magnetic resonance (CMR). **A** Systolic CMR cine frames derived from four- (*top row*), three- (*middle row*), and two-chamber (*bottom row*) LV views of a patient before and after transfemoral (TF) access (*left two columns*) as well as from a patient before and after transapical (TA) access (*right two columns*). The *green arrows* represent velocity vectors illustrating systolic inward motion. The TA transcatheter aortic valve implantation (TAVI) patient shows reduced systolic deformation of the apical LV segments 3 months after the procedure. **B** Average peak systolic radial strain values of 49 analyzed segments obtained from all TF-TAVI patients (*blue line*) and all TA-TAVI patients (*red line*). The apical segments are displayed in the *middle*, while the basal segments are displayed on the *left* and on the *right side* of the graph. There is a reduction in peak radial strain of the apical segments after TA-TAVI. Adapted with permission from Meyer et al. [58]

Interstitial and replacement myocardial fibrosis lead to impaired LV myocardial deformation which can be detected with strain imaging. Myocardial tagging and feature tracking CMR demonstrated that global as well as regional LV strains were significantly correlated with LGE extent in patients with hypertrophic cardiomyopathy, who exhibit a similar pattern of midwall fibrosis to patients with AS [52, 53]: global and regional LV strain values impair as LGE increases. Head-to-head comparisons between tagged and feature tracking CMR in moderate to severe AS have shown reasonable agreement for LV strain measurement, albeit feature tracking provided systematically higher values than CMR tagging [54, 55]. The correlation between CMR LV circumferential and longitudinal strain and strain rate and symptomatic status of patients with severe AS and preserved LV ejection fraction was demonstrated by Al Musa et al. [56]. LV longitudinal strain rate was the most sensitive parameter to discriminate between asymptomatic versus symptomatic patients (−106.3 ± 43.3%/s in patients with “no/mild” symptoms vs. −83.4 ± 24.8%/s in moderate and severely symptomatic patients; P = 0.048). The association between LV myocardial strain and outcomes after surgical or transcatheter treatment was demonstrated in two studies [36, 57]. Mahmod and coworkers showed that global LV circumferential, but not longitudinal strain measured on CMR significantly improved at 8 months after aortic valve replacement [57]. Similarly, LV circumferential strain by CMR tagging was significantly associated with all-cause mortality in 98 severe AS patients undergoing surgical and transcatheter aortic valve replacement (HR per each 1% deterioration of circumferential strain: 1.03; 95% CI 1.01–1.05; p = 0.009) [36]. Furthermore, the effect of procedural access (transfemoral vs. transapical) on LV mechanics was studied with CMR feature tracking in 44 patients undergoing transcatheter aortic valve replacement [58]. The transapical approach was associated with impaired peak systolic longitudinal strain of the apical segments as compared to the transfemoral approach (−8.9 ± 5.3 vs. −16.9 ± 4.3%, respectively; p < 0.001), while there were no differences in LV ejection fraction and peak systolic longitudinal strain of the basal and midventricular segments between both approaches (Fig. 7).

### Aortic regurgitation

**Fig. 8:**
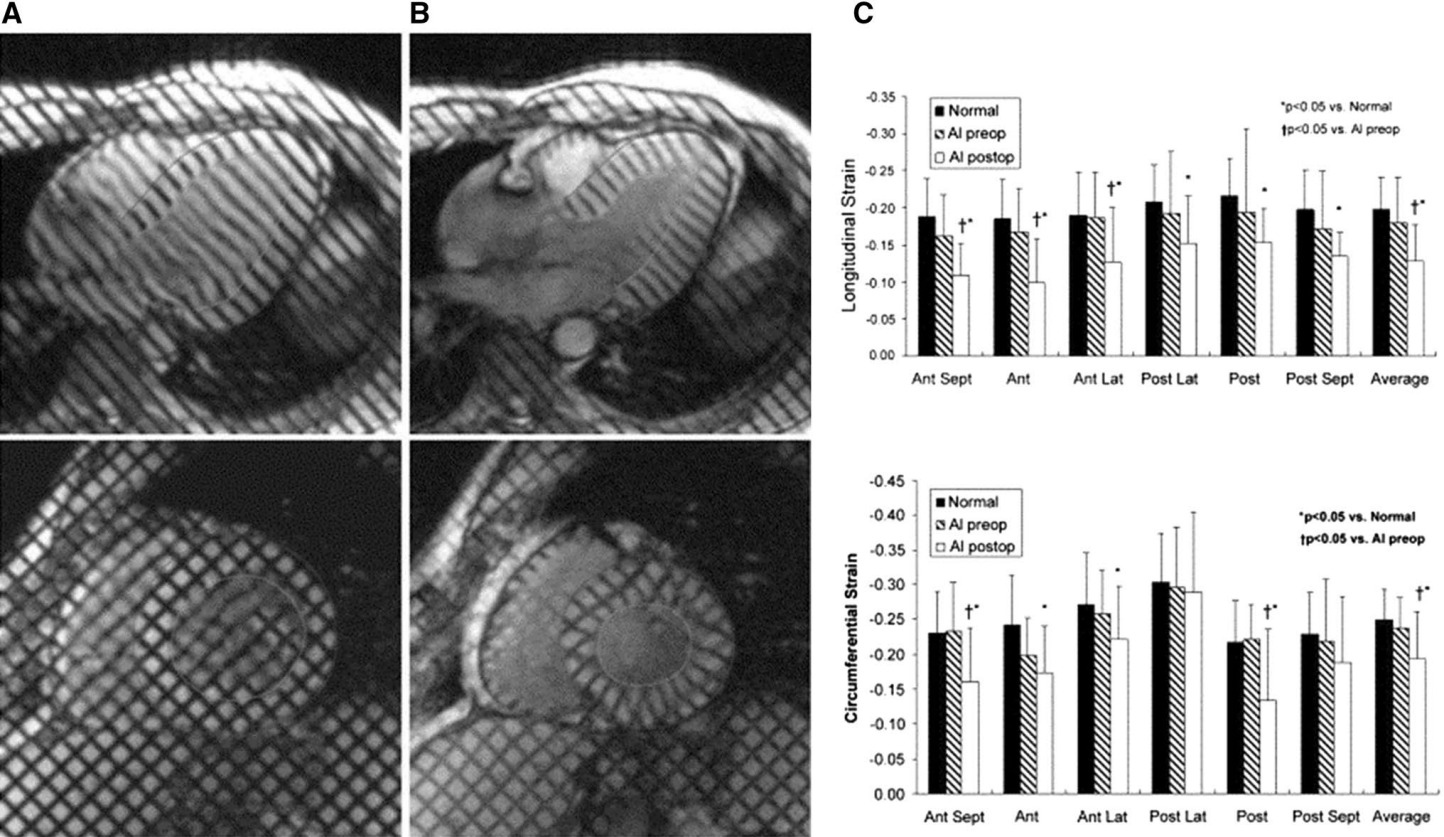
CMR tagging in patients with chronic severe aortic regurgitation. Left ventricular (LV) long-axis (*top row*) and short-axis (*bottom row*) cardiovascular magnetic resonance (CMR) tagging images at end-diastole (**A**) and at end-systole (**B**). A tagging pattern in the form of parallel lines was used for the long-axis cines and a grid pattern for the short-axis cines. Dedicated software was employed for the myocardial deformation analysis. **C** At an average of 28 ± 11 months after aortic valve replacement global and regional LV longitudinal and circumferential strain decreased (p < 0.05 for both global strain values) despite an improvement in LV ejection fraction and a decrease in LV size, which might imply an ongoing myocardial fibrosis after valve surgery. Adapted with permission from Pomerantz et al. [64]. *AI* aortic insufficiency, *Ant* anterior, *Lat* lateral, *Post* posterior, *preop* preoperative, *postop* postoperative, *Sept* septal

In aortic regurgitation (AR), pressure and volume overload induce growth of cardiomyocytes with addition of new sarcomeres in series and interstitial fibrosis, characterized by increased fibronectin and non-collagen components [59]. Several clinical studies have histologically proven pronounced myocardial fibrosis in severe AR at the time of valve surgery [37, 60, 61]. A few studies have also evaluated myocardial fibrosis with CMR [33, 62, 63]. Sparrow et al. compared myocardial T1 values measured with a modified Look-Locker technique before and after gadolinium contrast in eight patients with severe AR and 15 normal controls [62]. Patients with AR had significantly prolonged post-contrast T1 values in abnormally contracting segments compared to the controls (532 vs. 501 ms, respectively; p = 0.002), suggesting increased interstitial fibrosis. Furthermore, in nine patients with severe AR who underwent surgical aortic valve replacement, ECV measured on 3T CMR was strongly correlated with the extent of interstitial fibrosis on histology (r = 0.79, p = 0.011) [63]. Replacement fibrosis has been also described in 26 patients with severe AR by Azevedo et al. [33]. The authors reported a 69% prevalence of LGE, mostly following a multifocal pattern. The correlation between myocardial replacement fibrosis assessed with LGE and histopathology was good (r = 0.70, p < 0.001). Moreover, in a combined cohort of 26 patients with severe AR and 28 patients with severe AS, the amount of myocardial fibrosis was inversely correlated with LV functional improvement (r = −0.47; p = 0.02) and was associated with worse long-term survival after aortic valve replacement surgery (Chi square = 5.85; p = 0.02) (Fig. 6) [33]. Furthermore, in 14 patients with chronic severe AR, myocardial CMR tagging showed an impairment in global longitudinal and circumferential strain at 2 years after aortic valve replacement (p < 0.03 for both), despite an improvement in LV ejection fraction and a decrease in LV size (Fig. 8) [64]. Similarly, Ungacta et al. showed a decrease in posterior wall circumferential strain in patients with AR 6 months after valve replacement [65]. These findings suggest that the presence of LV myocardial fibrosis in patients with AR is a marker of adverse remodeling that may lead to further deterioration in LV strain and poor prognosis after aortic valve surgery.

### Mitral regurgitation

**Fig. 9:**
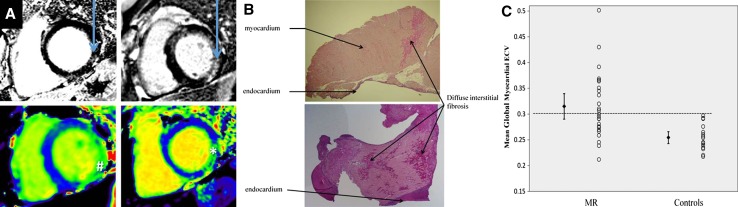
Cardiovascular magnetic resonance (CMR) myocardial fibrosis assessment in primary degenerative mitral regurgitation (MR). **A** Late gadolinium enhanced CMR images (*top*) and native T1 maps (*bottom*) in patients with MR. The *arrows* indicate the presence of midwall replacement fibrosis in the inferolateral wall. The native T1 values were increased in corresponding areas (*Hash* 1045 ms and *Asterisk* 1102 ms). **B** Left ventricular fibrosis demonstrated on histology: replacement fibrosis can be well-delineated (*upper plot*) or patchy (*lower plot*). **C** Individual patient data presented in the scatter plot demonstrate a wide overlap of the extracellular volume (ECV) values in patients with MR and controls. However, the mean and the standard error of the mean (*error bars*) were significantly larger in patients with MR as compared to the controls. Adapted with permission from Edwards et al. [68]

Mitral regurgitation (MR) is a heterogeneous disease, broadly classified as organic (primary) or functional (secondary) based on the underlying mechanism. Organic MR is due to intrinsic valvular disease whereas functional MR is caused by regional and/or global LV remodeling without structural abnormalities of the mitral valve [66]. Degenerative mitral valve disease (myxomatous disease and fibroelastic deficiency) is the most frequent etiology of primary MR in developed countries. The indication for mitral valve repair/replacement is determined by the presence of symptoms or LV function deterioration and LV remodeling [1, 3]. However, LV remodeling and myocardial fibrosis may occur before the development of symptoms. Chronic LV volume overload associated with MR leads to myocardial hypertrophy and increased interstitial fibrosis [67]. In 35 asymptomatic patients with moderate to severe primary MR, Edwards et al. demonstrated higher ECV on CMR as compared to controls (0.32 ± 0.07 vs. 0.25 ± 0.02, p < 0.01) (Fig. 9) [68]. Furthermore, 31% of patients with MR exhibited a non-infarct LGE pattern on CMR. Patients who had non-infarct type LGE presented with higher ECV values compared to MR patients without LGE (0.35 ± 0.02 vs. 0.27 ± 0.03, p < 0.01). The ECV values correlated with LV end-systolic volume, measures of systolic and diastolic LV dysfunction as well as with peak oxygen consumption on treadmill testing. The distribution of LGE in patients with MR varies significantly. Han et al. demonstrated the presence of LGE of the papillary muscles in 63% of patients with MV prolapse [69] whereas Chaikriangkrai et al. observed LV replacement fibrosis in 40% of patients with chronic severe MR [35]. The presence of LV LGE was associated with worse clinical outcomes in terms of intensive care unit readmission, incidence of permanent pacemaker implantation and rehospitalization (HR 4.775; 95% CI 1.100–20.729; p = 0.037) [35].

These structural changes of the LV myocardium may be associated with subtle functional abnormalities. In 15 patients with chronic moderate and severe MR and preserved LV ejection fraction who underwent CMR with tissue tagging, Maniar et al. demonstrated preserved global longitudinal and circumferential strain but abnormal regional strain values: the septal LV segments exhibited impaired strain whereas the lateral segments showed compensatory hyper-contractility [70]. Similarly, Mankad et al. showed with CMR tagging abnormal regional strain patterns in patients with severe MR and preserved LV ejection fraction: while radial strain was increased (19 ± 9 vs. 16 ± 6%, p = 0.003), circumferential strain was reduced (12 ± 6 vs. 21 ± 6%, p ≤ 0.001) as compared to healthy controls [71]. Several authors have demonstrated a decrease in global longitudinal and circumferential strain parameters on CMR tagging in patients with severe degenerative MR after mitral valve repair, which might imply an ongoing myocardial fibrosis after surgery [72–74].

## Future perspectives

Tissue characterization and strain imaging with CMR have provided new insights into the pathophysiology of VHD. Current guidelines recommend valve surgery in severe symptomatic VHD or when LV function decreases [1, 3]. However, early detection of LV structural and functional changes may help to identify patients who may benefit from early surgery. It is conceivable that early relief of the pressure or volume overload would result in less damage to the LV and better outcome at follow-up. However, there are currently no prospective data to evaluate whether early surgical valve treatment results in better prognosis in VHD. It may be challenging as well to define the cut-off values of ECV, T1 times, LGE and LV myocardial strains for therapeutic intervention. Standardization in data acquisition and analysis are important issues to be resolved.

The early valve replacement guided by biomarkers of left ventricular decompensation in asymptomatic patients with advanced aortic stenosis (EVOLVED) is the first multicenter randomized controlled clinical trial that will investigate whether the early valve intervention in patients with asymptomatic severe AS and midwall fibrosis on CMR improves patients’ clinical outcomes compared to the standard care (NCT03094143). The results of this study may have an impact on future guidelines and recommendations on treatment of VHD.

## Abbreviations

AR: Aortic regurgitation
AS: Aortic stenosis
CMR: Cardiovascular magnetic resonance
ECV: Extracellular volume
iECV: Indexed extracellular volume
LGE: Late gadolinium enhancement
MR: Mitral regurgitation
LV: Left ventricle/left ventricular
VHD: Valvular heart disease
